# Case report of treatment experience with idursulfase beta (Hunterase) in an adolescent patient with MPS II

**DOI:** 10.1016/j.ymgmr.2017.05.002

**Published:** 2017-05-11

**Authors:** Lock-Hock Ngu, Winnie Ong Peitee, Huey Yin Leong, Hui Bein Chew

**Affiliations:** Genetics Department, Hospital Kuala Lumpur, Malaysia

**Keywords:** Mucopolysaccharidosis, Asian, Enzyme replacement therapy, Idursulfase beta

## Abstract

Mucopolysaccharidosis (MPS) II or Hunter syndrome is a chronic, progressive, multi-systemic illness associated with significant morbidity and early mortality. Available evidence in Asian populations shows that Hunter syndrome has a mean age of onset of 2 to 5 years and a life expectancy of 13 years in more severely affected individuals, with respiratory failure reported as the leading cause of death. Enzyme replacement therapy (ERT) with idursulfase (Elaprase, Shire Pharmaceuticals) and idursulfase beta (Hunterase, Green Cross Corp) are the only approved treatment for patients with MPS II. While these agents have the same amino acids, they have different glycosylation patterns because they are produced in different cell lines via different manufacturing processes. In previous studies, the beneficial effects of idursulfase beta have been confirmed in patients up to 35 years of age, without serious treatment-related safety concerns. The major drawbacks associated with ERT include the potential development of serious infusion-related anaphylactic reactions and up to 50% of treated patients develop anti-IDS antibodies. Here we report the case of a 13-year-old Malaysian patient with attenuated MPS II who developed troublesome infusion-associated reactions while receiving idursulfase treatment but tolerated and responded favorably to idursulfase beta.

## 1 Introduction

Mucopolysaccharidoses (MPS) are hereditary, progressive diseases caused by mutations of genes coding for lysosomal enzymes leading to defects in stepwise breakdown of glycosaminoglycans (GAGs) [1]. GAGs, formally named mucopolysaccharides, are large, complex polymers of linear repeating sulfated acidic and amino sugar disaccharide units widely distributed in most of the tissues. The metabolic recycling of GAGs requires the stepwise degradation of the terminal sulfate, acidic, and amino sugar residues by a series of lysosomal enzymes. The deficiency of one of these enzymes blocks degradation of the substrate and results in a specific disorder.

MPS II or Hunter syndrome is inherited in an X-linked pattern [1], [2] with a reported incidence of 1.3 per 100,000 Caucasian male live births [1]. The deficiency or abnormality of iduronate-2-sulfatase (IDS) induces the accumulation of GAGs, dermatan sulfate and heparan sulfate, throughout body tissues and organs, such as the heart, liver, spleen, bones and joints. MPS II is the most common type of MPS in Asia; making up approximately 50% of all MPS types in countries such as Korea, Japan [3] and Taiwan [4].

The severe form of MPS II has a symptom onset of 2 to 4 years of age, with progression of somatic symptoms and severe cognitive impairment during childhood. Death most often occurs by 10 to 15 years of age [1], [5]. Clinical manifestations include severe airway obstruction, skeletal deformities, cardiomyopathy and, in most patients, neurological decline [2]. The diagnostic procedures in a male proband include quantitative and qualitative analysis of urinary GAGs excretion, and iduronate-2 sulfate sulfatase (IDS) enzymatic activity assay in enzyme activity in white cells, fibroblasts, or plasma. Documentation of normal enzymatic activity of at least one other sulfatase is required to exclude multiple sulfatase deficiency, which can share some common clinical features with MPS II and IDS enzymatic activity is also reduced. The mutation analysis is necessary for the proper genetic counseling. The knowledge of molecular background of the disease in the affected family is also useful in detection of female carriers. However, it is difficult to establish a genotype-phenotype correlation to provide an indication of the likely prognosis. This is because individuals carrying the same alterations may present with different phenotypes, suggesting that other factors may modulate the clinical phenotype [2].

**Fig. 1:**
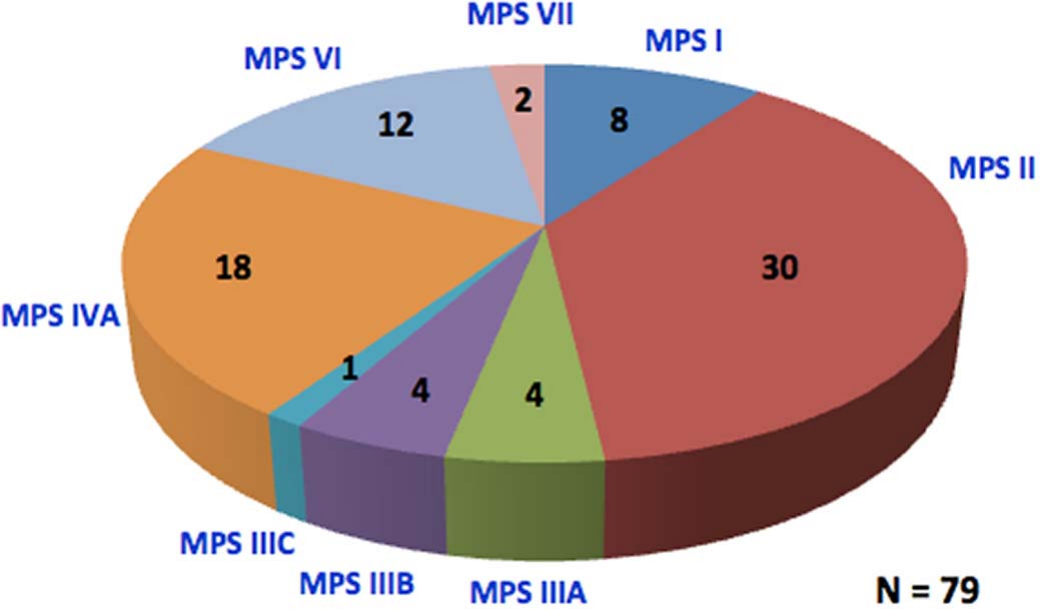
Prevalence of mucopolysaccharidoses (MPS) at the National Referral Centre, Genetics Department, Hospital Kuala Lumpur, Malaysia.

There are limited epidemiological data for MPS in the Malaysian population; however, available data from the National Referral Centre at Hospital Kuala Lumpur, Malaysia suggest that the majority of patients (38%) have MPS II (Fig. 1).

Until a decade ago, there was no effective therapy for MPS II, and care has largely been palliative with a focus on signs and symptoms [1]. Hematopoietic stem cell transplantation (HSCT) has variable success in patients with MPS II; the high morbidity and mortality associated with HSCT and the lack of clear evidence supporting neurocognitive benefits have limited its use. Enzyme replacement therapy (ERT) is the only approved treatment for patients with MPS II: idursulfase (Elaprase, Shire Pharmaceuticals) and idursulfase beta (Hunterase, Green Cross Corp) are available as a weekly intravenous (IV) infusion at 0.5 mg/kg body weight over 1 to 3 h. ERT have been shown to improve many of the signs and symptoms, and overall wellbeing of MPS II patients.

These two agents have the same amino acids but different glycosylation patterns because they are produced in different cell lines via different manufacturing processes: cell line sources are CHO cells for idursulfase beta and transformed human cells (HT-1080) for idursulfase [6]. Idursulfase beta is a glycoprotein containing 525 amino acids (2 disulfide bonds) and 8 N-linked glycosylation sites that are occupied by complex, hybrid, and high-mannose type of oligosaccharide chains [2]. In addition, idursulfase beta exhibited significantly higher specific enzyme activity than idursulfase as a result of its higher formylglycine (FGly) content [7]. In a comparative study on patient fibroblasts and a murine model, idursulfase beta exhibited enhanced in vitro efficacy at a lower drug concentration and enhanced in vivo efficacy with regards to the degradation of tissue GAGs and improvement of bone, with reduced anti-drug antibody formation [6]. Clinical trial data in patients aged < 6 years [8] and those aged 6–35 years [9], confirm the efficacy of idursulfase beta in significantly reduced urinary GAGs in patients treated for up to 53 weeks and improved the 6-minute walk test, without serious treatment-related safety signals. The major drawbacks associated with ERT include the potential development of serious anaphylactic reactions up to 24 h after infusion; up to 50% of treated patients develop anti-IDS antibodies, which may limit long-term product efficacy, and the inability of recombinant IDS to cross the blood-brain barrier and reach the central nervous system following IV delivery, thus limiting the potential applicability to treat neurological symptoms [10].

Here we report the use of idursulfase beta in a 13-year-old Malaysian patient with MPS II.

## 2 Case history

The patient is the elder of 2 boys of non-consanguineous parents. At 21 months of age, he presented with frequent nasal congestion, snoring at night and progressive hepatomegaly. A cardiac murmur due to mitral and aortic regurgitation as well as progressive joint contractures was detected when the patient was aged 4 years. He was diagnosed with MPS II at the age of 6 years, based on an elevated urinary GAG of 34.45 mg/mmol creatinine (reference range < 11 mg/mmol creatinine) with raised dermatan and heparan sulfate, and undetectable iduronate-2-sulfatase activity in peripheral blood leukocytes. Molecular confirmation revealed a c.1608_1609delTA (p.Tyr536Ter) mutation in exon 9 of the *IDS* gene. This novel frameshift mutation leads to early termination of the amino acid coding, which is expected to affect the function of IDS enzyme.

At 11 years of age, his weight and height were at the third percentile at 26.3 kg and 126 cm, respectively. Head circumference was 55 cm which is at the 97th percentile. The liver was palpable 5 cm below the right costal margin measured at the midclavicular line; the spleen was not palpable. A sleep study showed mild obstructive sleep apnea. Echocardiography showed moderate aortic regurgitation with thickened aortic valve and mild mitral regurgitation and his 6-minute walk test was 440 m. Ophthalmology assessment showed no corneal clouding and no other abnormality. Audiology assessment, using tympanometry and pure tone audiometry revealed normal hearing bilaterally. An IQ assessment using the Wechsler Preschool and Primary Scale of Intelligence (WPPSI) showed an average IQ of 97. A spinal MRI did not show any spinal stenosis.

## 3 Treatment course

**Fig. 2:**
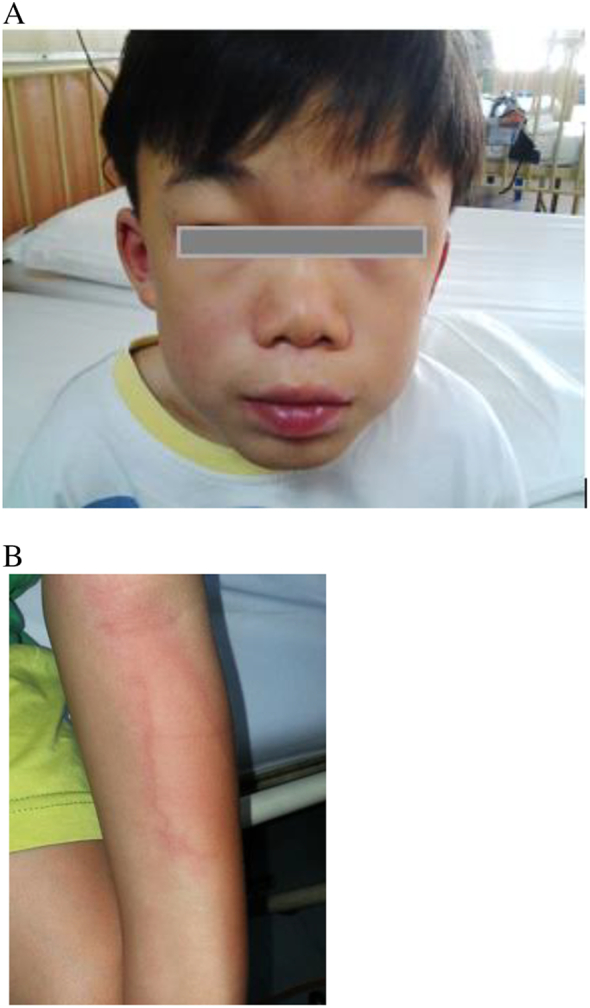
Clinical features of infusion-associated reaction with IV idursulfase in the patient. (A) Swollen eyes and lips with erythematous rash on the face (B) Urticarial rash of the left forearm

When the patient was aged 11 years, treatment was initiated with idursulfase (Elaprase) at 0.5 mg/kg (12 mg) over 4 h with pre-medication of intravenous hydrocortisone 4 mg/kg, promethazine 10 mg and oral loratadine 10 mg. The weekly infusions were initially well tolerated. However, he developed an adverse drug reaction, presenting with generalized urticarial rash on the 26th infusion. Idursulfase was stopped for 1 h and recommenced when the rash had diminished. During the next infusion 2 weeks later, the patient again developed generalized urticaria despite starting at a slower infusion rate. The idursulfase dose was subsequently reduced to 0.25 mg/kg (6 mg) over 6 h on the 28th infusion. The 47th infusion was not completed due to recurrent urticarial rash and hypotension during infusion despite treating with additional intravenous chlorpheniramine. Treatment was discontinued for 2 weeks and re-commenced at 1 mg weekly; this dose was slowly increased but only to a maximum of 6 mg infused over a period of 6 h plus premedication with oral prednisolone, cetirizine, chlorpheniramine and promethazine. Localized urticaria developed over both of the patient's upper limbs towards the end of the infusion. After the patient had received approximately 100 infusions of idursulfase, it was established that any dose > 6 mg would be associated with generalized urticaria (Fig. 2a and b).

The patient showed minimal clinical improvement during this period due to sub-optimal treatment. Serum anti-idursulfase IgG was detected at a titer of 1:400 but serum anti-idursulfase immunoglobulin (Ig) E was not detected. Urinary GAGs remained elevated at a range of 19.74–52.64 mg/mmol creatinine (Table 1) due to the suboptimal dose and frequent interruption of ERT.

**Table 1:** Clinical assessment prior to ERT and after idursulfase (Elaprase) and idursulfase beta (Hunterase) treatment.

Examination/investigation	Before commencement of ERT	After 24 months of Idursulfase treatment	After 20 months of idursulfase beta treatment
Weight, kg	26.3 (3rd centile)	28 (< 3rd centile)	36 (< 3rd centile)
Height, cm	126 (3rd centile)	133 (< 3rd centile)	144.5 (< 3rd centile)
Head circumference, cm	55 (97th centile)	54 (50–98th centile)	54 cm (50–95th centile)
Liver and spleen size	Liver palpable 5 cm below right costal margin at the midclavicular line No spleen palpable	Liver and spleen not palpable	Liver and spleen not palpable
Sleep study/overnight pulse oximetry study	Mild obstructive sleep apnea	No significant desaturation during sleep	No desaturation during sleep
Echocardiography	Mild mitral regurgitation. Thickened aortic valve with moderate aortic regurgitation Dilated left atrium and left ventricle	Mild mitral and aortic regurgitation with thickened aortic valve Dilated left atrium and left ventricle	Mild mitral and aortic regurgitation Left atrium and left ventricle not dilated
6-minute walk test	440 m	460 m	515 m
Ophthalmology assessment	No corneal clouding and no other abnormality	No corneal clouding Eye test with Snellen chart bilaterally 6/6	No corneal clouding Eye test with Snellen chart bilaterally 6/6
Hearing assessment	Normal hearing bilaterally (using tympanometry and pure tone audiometry)	Normal bilaterally (using pure tone audiometry)	Normal bilaterally (using pure tone audiometry)
Cognitive function	Using WISC-IV (Weschler intelligence scale for children-4th edition) - average IQ of 97	Final exam report (total 9 subjects) - 2C, 3D and 4E	Final exam report (total 9 subjects) - 3C, 5D and 1E
Urine GAG, mg/mmol creatinine	34.6–50.6	19.74–52.64	12.17–26.1

**Fig. 3:**
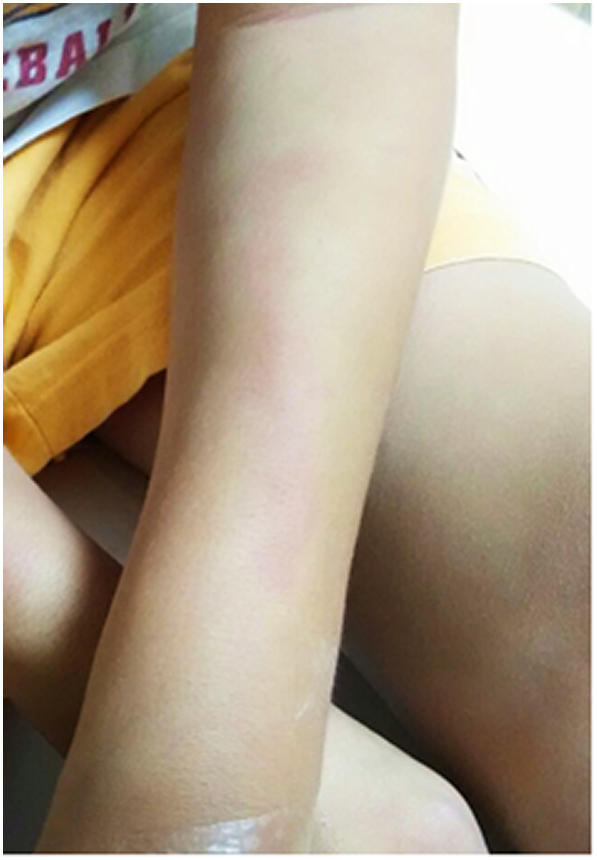
Milder skin rash tracking along the infusion site on the left forearm with IV idursulfase beta.

**Fig. 4:**
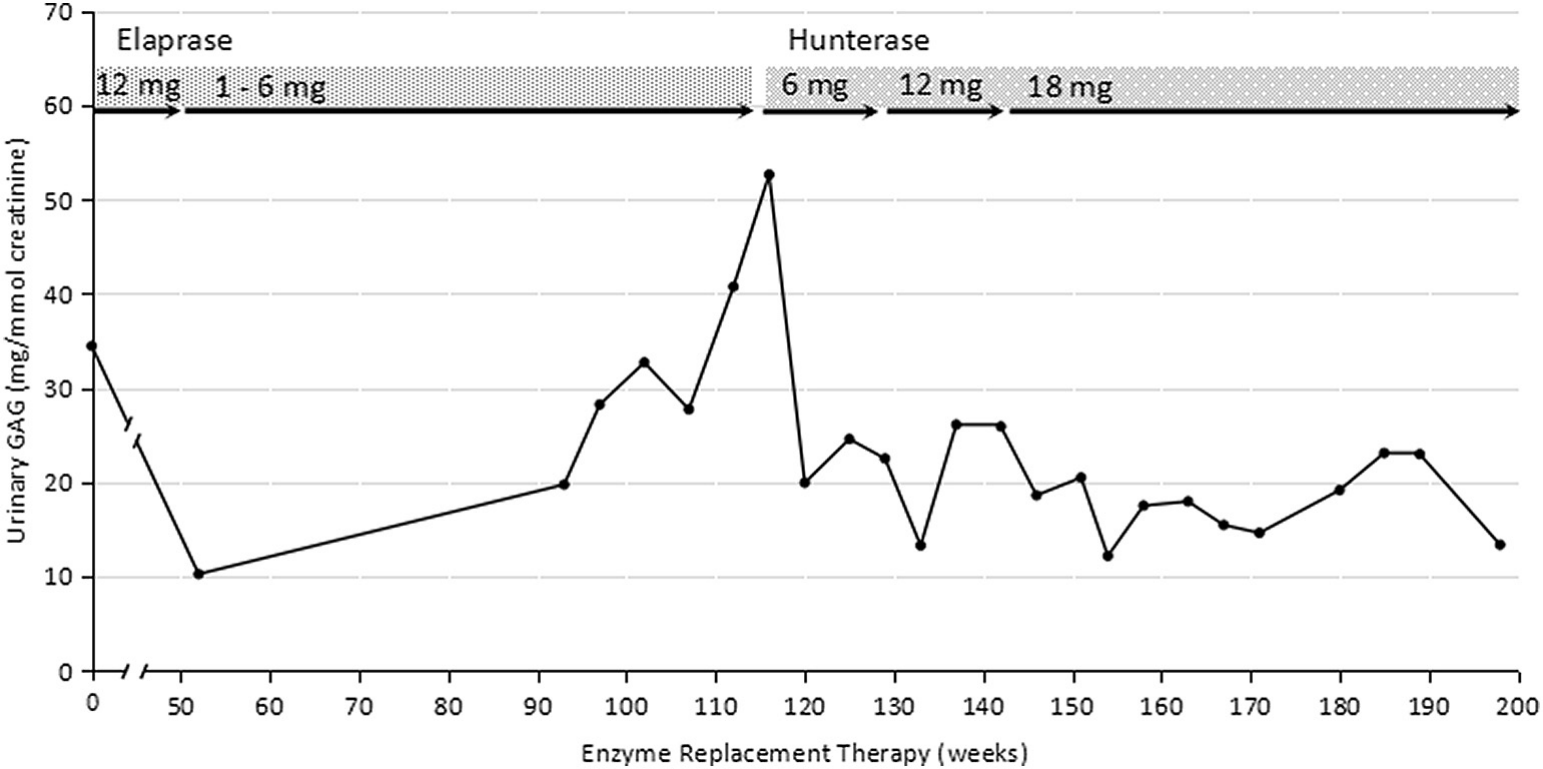
Urine GAG levels during ERT. Urinary GAGs were high during suboptimal doses of IV idursulfase (Elaprase) when patient developed intolerable adverse drug reactions and urinary GAGs were low during optimal treatment dose with IV idursulfase beta (Hunterase) (18 mg = 0.5 mg/kg body weight).

At 13 years old, he was switched to weekly idursulfase beta (Hunterase) infusions; the dose was gradually increased from 6 mg/infusion to 18 mg/infusion (0.5 mg/kg/week). After 20 months of treatment with idursulfase beta, the urine GAGs declined to 12.17–26.1 mg/mmol creatinine, anti-idursulfase IgG was no longer detectable and there was no antibody against idursulfase beta. To date, the patient had received approximately 80 idursulfase beta infusions. The infusions were well tolerated; with only an occasional mild rash over the infusion site (Fig. 3). Infusion time had decreased to about 4 to 5 h. The patient's symptoms remained stable, he continued to gain both weight and height, and his endurance was much improved. Urine GAGs further decreased (Table 1, Fig. 4).

## 4 Discussion

The management of children and adolescents with MPS II presents a significant clinical challenge not only to healthcare providers but also to families and caregivers [11]. Caring for patients with a chronic, progressive and degenerative disorder, such as MPS II, has an impact on all dimensions of family life. Therefore, the approval of ERT for the treatment of MPS II provides a promising therapeutic strategy with which to manage this patient population. Idursulfase has been approved by National Pharmaceutical Regulatory Agency of Malaysia for the treatment of MPS II in 2007. To date, 13 MPS II patients have been treated with idursulfase at our centre. We have obtained special approval from the agency to use idursulfase beta for the patients in this case report.

Recombinant enzymes, idursulfase beta (Hunterase) and idursulfase (Elaprase), are currently available for ERT: these two enzymes have 100% identical amino acid sequences derived from the human IDS gene (NM_000202). However, these two enzymes can be differentiated based on a number of characteristics, including differing product cell lines (CHO-DG44; idursulfase beta vs HT-1080; idursulfase), cultivation (serum-free vs with serum, respectively), % formylglycine (79.40 vs 68.12, respectively) and K_uptake_ (5.09 vs 6.50 nM, respectively). Although these enzymes have the same amino acids, they have different glycosylation patterns and glycosylation is a crucial factor in the targeting process for ERT of lysosomal enzymes, which is governed by receptor-mediated uptake. Idursulfase beta exhibited faster time-dependent uptake into patient's fibroblasts [6].

IgE-mediated anaphylaxis and allergic reactions have recently been reported with idursulfase and antibody formation is reported to occur within 4 to 8 weeks [6]. True anaphylaxis due to an IgE-mediated response has not been observed as frequently; although it can occur at any time it would be expected to occur early in the infusion. Therefore, premedication with an antihistamine is primarily used to prevent IgE-mediated histamine release and to help mitigate a reaction if one were to occur. Symptoms may include flushing, rash, swelling of lips/tongue/uvula, respiratory compromise (e.g., dyspnea, wheeze-bronchospasm, stridor, reduced peak expiratory flow, hypoxemia), reduced blood pressure or associated symptoms (hypotonia, syncope, incontinence), and persistent gastrointestinal symptoms (e.g., abdominal cramps, vomiting).

Anaphylactoid reactions, the most important severe type of infusion-associated reactions, are caused by synchronous complement activation due to IgG antibodies to the enzyme and most commonly occur during the first hour after the infusion rate is increased. In MPS VII mice, these types of reactions were observed approximately 4–6 weeks into infusions [12]. The symptoms can include fever, chills, respiratory distress, tachycardia, nausea and abdominal pain/cramps. A slow rate of infusion for 1 h followed by the increased rate of infusion is expected to reduce complement-mediated hypersensitivity responses, based on studies with laronidase in MPS I [13].

Urticarial skin reactions with or without angioedema (tongue and throat swelling) are a common reaction often observed after several months of therapy. Whether the underlying pathophysiology of these reactions are IgE or IgG mediated has been debated in the literature. Regardless of its cause, these symptoms are associated with significant pruritus and discomfort for the patient. Increasing the premedication and slowing the infusion rate can help. These types of reactions tend to subside over a period of weeks to months with continued therapy. Airway obstruction is potentially serious in patients with MPS and floppy tracheal cartilage, so the occurrence of angioedema demands particular care, caution and appropriate management. Angioedema should be immediately addressed by stopping the infusion and giving appropriate doses of subcutaneous epinephrine, intravenous diphenhydramine, and hydrocortisone or methylprednisolone; institution of positive-airway pressure may be needed. Fortunately, acute airway complication during ERT is rare in patients with MPS II. In this case report, the patient did not experience angioedema. He also did not develop worsening airway obstruction. However, recurrent urticarial skin reaction which caused significant discomfort necessitated a change in the treatment course.

The definition of effective treatment for MPS II is an improvement in or a prevention of progression of disease activity as indicated by a stabilization in clinical condition associated with an improvement in the abnormalities present at baseline [14]. The patient benefited from ERT with idursulfase beta with clinically encouraging growth, significant reduction of urinary GAGs and an improvement in endurance, as measured by 6-minute walk test. Importantly, this case report highlights the tolerability and safety of idursulfase beta in a patient with MPS II after persistent intolerable adverse drug reactions with idursulfase. Switching from idursulfase to idursulfase beta enabled optimization of his treatment and allowed the patient to maintain his quality of life.

The immunogenicity of a recombinant enzyme depends on multiple factors including, but not limited to, the characteristics and status of the immune system of an individual patient, the origin of the producer cell line, differences in the cell culture and purification processes, the protein structure and post-translational modifications of the recombinant protein. The producer cell line of idursulfase (HT-1080) was created from tissue taken in a biopsy of a fibrosarcoma present in a 35-year-old human male [15]. HT-1080 cells may show different glycosylation patterns of cellular proteins compared with those in non-cancer cells [16], which may result in altered immune responses, although the proteins were produced in human cells. In addition, for idursulfase, animal derived materials such as bovine serum are used in the processes of cell bank manufacturing and fermentation. In contrast, idursulfase beta is manufactured without any animal or human serum. This is because CHO cells can grow in serum-free and chemically defined media which ensures reproducibility between different batches of cell culture [17]. Different manufacturing processes and cell line sources may contribute to the differences in the immune response to idursulfase and idursulfase beta.

Both idursulfase beta and idursulfase contain complex, hybrid and high-mannose types of oligosaccharide chains but idursulfase has a relatively higher level of the mannose type [6]. The mannose part is recognized by the mannose receptor (a highly endocytic receptor) of the immune cell, macrophage, which causes the macrophage to pull the protein into the cell via endocytosis. Proteins brought into macrophages are degraded and processed to short peptide, which are presented by MHC class I or II molecules on the cell surface. If the exposed peptides are not recognized as self-antigens by immune cells, such as T or B cells, it can lead to antibody production by B cells. Therefore, the low proportion of high-mannose type glycosylation in idursulfase beta may possibly contribute, to some extent, to its better immunogenicity profile relative to that of idursulfase. In a comparative study on idursulfase and idursulfase beta, the cellular enzyme uptake activity for idursulfase beta was significantly higher than that for idursulfase [7]. Higher cellular uptake means that the idursulfase beta remains in the blood for a shorter time reducing the risk of recognition by immune cells, thereby possibly lessening the immune response.

Our patient does not have significant central nervous system (CNS) involvement. He has demonstrated improvement in indicators of somatic disease following ERT. However, the majority of MPS II patients have progressive CNS disease. Both idursulfase and idursulfase beta administered intravenously do not cross the blood–brain barrier and therefore are not expected to have a significant effect on the neurological features of MPS II. Hematopoietic stem cell transplantation (HSCT) has been used as a potential alternative. However, anecdotal case reports published to date are unable to show clear evidence that HRCT treatment early in life significantly reduces the progression of neurologic disease in MPS II. Furthermore, HSCT is associated with a high risk of morbidity and mortality. Research and development of other CNS-directed therapeutic options is needed to address this unmet need in MPS II patients with the severe form of disease.

## Conflict of interest

The authors declare that they have no conflict of interest.

## Funding

This research did not receive any specific grant from funding agencies in the public, commercial, or not-for-profit sectors.

## Disclosures

L.H.N., W.O.P., H.Y.L., and H.B.C. have received honoraria from BioMarin and Genzyme. L.H.N. and W.O.P. were involved in a clinical trial sponsored by Alexion and Shire. H.Y.L. was involved in clinical research sponsored by Alexion and BioMarin and H.B.C. was involved in research sponsored by Alexion, BioMarin and Shire.
